# Novel method for production and purification of untagged pneumococcal surface protein A from clade 1

**DOI:** 10.1007/s00253-024-13098-2

**Published:** 2024-04-04

**Authors:** Tasson da Costa Rodrigues, Patricia Zorzete, Eliane Namie Miyaji, Viviane Maimoni Gonçalves

**Affiliations:** 1 Laboratório de Bacteriologia, Instituto Butantan, São Paulo, São Paulo, Brazil; 2 Programa de Pós-Graduação Interunidades Em Biotecnologia, Universidade de São Paulo, São Paulo, São Paulo, Brazil; 3 Laboratório de Desenvolvimento de Vacinas, Instituto Butantan, São Paulo, São Paulo, Brazil

**Keywords:** *Streptococcus pneumoniae*, PspA, Recombinant protein, Purification, Antigen, Downstream processing

## Abstract

**Abstract:**

*Streptococcus pneumoniae* can cause diseases with high mortality and morbidity. The licensed vaccines are based on capsular polysaccharides and induce antibodies with low cross reactivity, leading to restricted coverage of serotypes. For surpassing this limitation, new pneumococcal vaccines are needed for induction of broader protection. One important candidate is the pneumococcal surface protein A (PspA), which can be classified in 6 clades and 3 families. We have reported an efficient process for production and purification of untagged recombinant PspA from clade 4 (PspA4Pro). We now aim to obtain a highly pure recombinant PspA from clade 1 (PspA1) to be included, together with PspA4Pro, in a vaccine formulation to broaden response against pneumococci. The vector pET28a-*pspA1* was constructed and used to transform *Escherichia coli* BL21(DE3) strain. One clone with high production of PspA1 was selected and adapted to high-density fermentation (HDF) medium. After biomass production in 6 L HDF using a bioreactor, the purification was defined after testing 3 protocols. During the batch bioreactor cultivation, plasmid stability remained above 90% and acetate formation was not detected. The final protein purification process included treatment with a cationic detergent after lysis, anion exchange chromatography, cryoprecipitation, cation exchange chromatography, and multimodal chromatography. The final purification process showed PspA1 purity of 93% with low endotoxin content and an overall recovery above 20%. The novel established process can be easily scaled-up and proved to be efficient to obtain a highly pure untagged PspA1 for inclusion in vaccine formulations.

**Key points:**

• *Purification strategy for recombinant PspA1 from Streptococcus pneumoniae*

• *Downstream processing for untagged protein antigens, the case of PspA1*

• *Purification strategy for PspA variants relies on buried amino acids in their sequences*

**Supplementary Information:**

The online version contains supplementary material available at 10.1007/s00253-024-13098-2.

## Introduction

One of the most important human pathogens, *Streptococcus pneumoniae* or pneumococcus, can colonize the nasopharyngeal microbiota without causing any symptoms. But in some cases, it can cause severe diseases with high mortality and morbidity, especially in the population under 5 years of age and the elderly (WHO - World Health Organization 2019). As an example, in 2019, the pneumococcus was associated to 829,000 causes of death worldwide (GBD 2019 Antimicrobial Resistance Collaborators 2022). Lower respiratory tract infections (LRTI) caused 3.7 million of severe disease cases in 2015 (Wahl et al. 2018) and pneumococcal pneumonia was associated with 55.4% of all deaths from LRTI (Troeger et al. 2017). This bacterium has a capsule composed of polysaccharide (PS), its major virulence factor. Based on the chemical structure and serum recognition, the PS can be classified in more than 100 serotypes, and the number of serotypes may be underestimated because of the limitations of traditional serotyping methods (Ganaie et al. 2023; Pimenta et al. 2021).

The most important way to control pneumococcal diseases is vaccination of the population at risk. There are currently two types of vaccines licensed, but both are based on the induction of antibodies against the PS, with poor cross protection between serotypes. The first generation of pneumococcal vaccines was produced with PS from the 23 most frequently isolated serotypes in pneumococcal invasive disease (IPD). The immune response induced is T cell independent and because of that, it is not long lasting (Huss et al. 2009; WHO - World Health Organization 2008). The second generation was based on the conjugation of the PS to carrier proteins, which induced a T cell response and made possible the immunization of children (Kaplan et al. 2002; WHO - World Health Organization 2007). After the introduction of the pneumococcal conjugate vaccines (PCV), the number of cases of IPD reduced drastically, especially in children under 5 years and in disease caused by vaccine serotypes (VT) (Moreira et al. 2016; Wu et al. 2015). Another observation reported in the post-PCV era was the induction of protection for non-vaccinated individuals by herd effect (Barnett et al. 2015; Bechini et al. 2009). On the other hand, serotype replacement by non-vaccine serotype (NVT) was observed after PCV introduction (Andrade et al. 2016; Brandileone et al. 2019). The protection against non-invasive disease induced by the PCV still is a point of controversy (Hu et al. 2023; Loo et al. 2014; Nakano et al. 2020).

Some researchers are now focusing on the development of a novel generation of pneumococcal vaccines, which can induce a serotype independent response. Together with the inactivated whole cell vaccine, some pneumococcal antigens have been studied as vaccine candidates, such as pneumococcal surface protein A (PspA), pneumococcal surface protein C (PspC), pneumolysin (Ply), and pneumococcal surface antigen A (PsaA) (Oliveira et al. 2021). Among these proteins, PspA is an important antigen candidate to be included in the PCVs or as a stand-alone antigen, which can induce protection against both colonization and invasive disease (Briles et al. 2001; Colichio et al. 2020; Miyaji et al. 2013). PspA plays an important role in the pathogenesis of pneumococci by avoiding complement deposition on the surface of the bacteria and inhibiting the bactericidal activity of apolactoferrin (Ren et al. 2004; Shaper et al. 2004; Tu et al. 1999). PspA is present in all clinical isolates and its molecular weight varies from 67 to 99 kDa. The mature molecule can be divided as α-helix N-terminal domain exposed outside the capsule, proline-rich domain, one domain with a repetition of 20 amino acids, and a C-terminal domain, which is responsible to anchor the protein on the cell wall (Yother and Briles 1992; Yother and White 1994). The final portion of the N-terminal domain, right before the proline-rich domain, named as clade-defining region (CDR), shows variability in the amino acid sequence. Based on that, PspA can be classified in 6 clades clustered in 3 families. Family 1 of PspA includes clades 1 and 2; family 2 includes clades 3, 4, and 5; and family 3 includes only clade 6. Proteins from the same clade share more than 90% similarity in the CDR. Those with similarity of the CDR around 72% are clustered in the same family, but in different clades. When the similarity of the CDR is less than 50%, the proteins are separated in different clades and families (Hollingshead et al. 2000). The distribution analysis of the families indicates that families 1 and 2 are the most frequent in clinical isolates. The two families are present in 80 to 99.7% of isolates, depending on the location of the study (Chang et al. 2021; Hollingshead et al. 2006, 2000). In previous work from our group, the mature N-terminus and the proline-rich domains of PspA from different clades of families 1 and 2 were cloned and expressed as recombinant proteins. The proteins containing a His-tag were purified by affinity chromatography and used to immunize mice. Recombinant PspA from clade 4 (PspA4Pro) showed good protection against both families, whereas recombinant PspA from clade 1 (PspA1) showed recognition and protection more restricted to the same family. Proteins were not assessed for final purity and endotoxin content though (Moreno et al. 2010).

The process for purification of untagged PspA4Pro had been described before by our group (Figueiredo et al. 2017). In the work, PspA4Pro was produced and purified using a combination of chromatography resins. Different conditions were also applied to increase recovery of the protein. The final fraction recovered showed protein purity of almost 98%. We also used this highly pure PspA4Pro for the development of a nanoparticle vaccine that induced good protection against strains expressing proteins from the same family but did not protect against strains expressing PspA from different families (Figueiredo et al. 2022; Rodrigues et al. 2018). We concluded that, for achieving broad coverage, the vaccine must induce response against the two major families of PspA. For that reason, a protein from family 1 of PspA must be cloned, produced, and purified for inclusion in a new vaccine formulation. Here, we demonstrate that the differences in the amino acid sequence of PspA1 and PspA4Pro can interfere in the purification protocol, inducing changes in each step and also leading the need of one final step to concentrate the protein and to reach protein purity above 90%, defined by the World Health Organization (WHO) for the carrier proteins used in the PCVs (WHO - World Health Organization 2009).

## Material and methods

### Obtaining a clone expressing PspA1

The *pspA1* gene from *S. pneumoniae* strain St 435/96 (kindly provided by Dr. Maria Cristina C Brandileone from Instituto Adolfo Lutz, Brazil) was stored as plasmid pAE-*pspA1*, produced by Moreno et al. (2010). pAE-*pspA1* has an insert of 1020 bp of *pspA1* (GenBank AY082387.1). This plasmid was used as template for amplification of the sequence encoding the N-terminal and proline-rich domains by polymerase chain reaction (PCR) using primers PspA1 pET F w/o His (5′ TAGCCATGGAAGAAGCGCCCGTAGC 3′) and PspA1 pET R w/o His (5′ TAGCTCGAGTTATGGTTGTGGTGCTGAAGC 3′). The fragment was then inserted into plasmid pGEM® T Easy (Promega, USA). The ligation reaction was used to transform strain *Escherichia coli* DH5α (Invitrogen, USA). Clones were selected in lysogenic broth with 100 mg/L ampicillin (LB-Amp) and plasmids were purified using QIAprep Spin Miniprep Kit (QIAGEN, USA). Cloning was confirmed by sequencing. Both the purified plasmid and the commercial plasmid pET-28a ( +) (Novagen, USA) were digested with the enzymes *Nco* I (Thermo Fisher Scientific, USA) and *Xho* I (Invitrogen, USA). The *pspA1* fragment was inserted into pET-28a using a T4 ligase (Invitrogen, USA) reaction at 4 °C for 16 h, and the ligation reaction was used to transform *E. coli* strain DH10β (Invitrogen, USA). Clones were selected in LB with 50 mg/L kanamycin (LB-Kan) and plasmids were purified using QIAprep Spin Miniprep Kit (QIAGEN, USA). For confirmation of the construction, different clones were digested with *Nco* I and *Xho* I. One clone was used to transform *E. coli* strain BL21(DE3) (Invitrogen, USA) for expression of PspA1. For confirmation of the gene expression, clones were then cultivated in 10 mL LB-Kan at 37 °C and 250 rotations per minute (rpm) agitation. The optical density at wavelength of 600 nm (OD_600nm_) of the culture was measured. When the OD_600nm_ reached 0.6, 1 mL sample was collected and the inducer isopropyl-β-D-thiogalactopyranoside (IPTG) was added to a final concentration of 0.1 mM. The culture was continued for 3 h, when another 1 mL sample was collected. Both, before and after IPTG, samples were centrifuged at 17,696 × g for 2 min and pellets were resuspended with SDS buffer with 2-mercaptoethanol. PspA1 expression was then confirmed by 12% SDS-PAGE.

### High-density fermentation medium preparation

HDF medium was prepared as described by Riesenberg et al. (1991) with some modifications. The content of the broth included 30.30 mM (NH_4_)_2_HPO_4_ (diammonium hydrogen phosphate), 97.79 mM KH_2_PO_4_ (potassium phosphate monobasic), 8.10 mM HOC(COOH)(CH_2_COOH)_2_·H_2_O (citric acid monohydrate), 0.41 mM C_6_H_5_FeO_7_ (ferric citrate), 0.02 mM CoCl_2_ (cobalt dichloride), 0.08 mM MnCl_2_·4H_2_O (manganese(II) chloride tetrahydrate), 0.01 mM CuCl_2_·2H_2_O (cupric chloride dihydrate), 0.05 mM H_3_BO_3_ (boric acid), 0.01 mM NaMoO_4_·2H_2_O (sodium molybdate dihydrate), 0.15 mM Zn(CH_3_COO)_2_·2H_2_O (zinc acetate dihydrate), 0.05 mM (HO_2_CCH_2_)_2_NCH_2_CH_2_N(CH_2_CO_2_H)_2_ (EDTA), 1.42 mM MgSO_4_·7H_2_O (magnesium sulfate heptahydrate), and 0.01 mM C_12_H_17_ClN_4_OS·HCl (thiamine hydrochloride). Glucose was used as carbon source at concentration of 55.51 mM. The culture was carried out with the plasmid selection marker C_18_H_36_N_4_O_11_·H_2_O_4_S (kanamycin) (HDF-Kan) at concentration of 0.09 mM and the pH was corrected to 6.7. The anti-foaming agent polypropylene glycol was added during the cultivation to a final concentration of 0.03% (w/v).

### Preparing the stock of the PspA1 producing clone and bioreactor cultivation

*E. coli* BL21(DE3) strain transformed with pET-*pspA1* was plated on M9 minimal (Sigma-Aldrich/Merk, Germany) agar supplemented with kanamycin and cultivated at 37 °C for 24 h. One colony from the plate was transferred to 10 mL M9 minimal (Sigma-Aldrich/Merk, Germany) broth supplemented with kanamycin (M9-Kan), cultivated for 16–18 h at 37 °C and 250 rpm. The culture was then transferred to 50 mL M9-Kan broth in a 500-mL flask. The 50 mL culture starting point was obtained by transferring a volume from the first flask to an initial OD_600nm_ of 0.1 and the culture was carried out at 37 °C and 250 rpm. When the OD_600nm_ reached 0.4, the culture was recovered and centrifuged at 2396 × g for 30 min. The seed stock was prepared by concentrating 10 times the transformed strain in M9-Kan supplemented with 15% (v/v) glycerol and stored at − 80 °C.

For the batch cultivation in a 10-L bioreactor, we used similar parameters as described before by Carvalho et al. (2012) and Figueiredo et al. (2017). The Biostat C-Plus (Sartorius, Germany) controlled by the MFCS/win 3.0 software (Sartorius, Germany) was filled with 6 L of HDF-Kan. The bioreactor culture was inoculated by transferring the volume from a culture prepared with the seed stock in HDF-Kan 16 h before at 30 °C and 300 rpm. The culture was started with an initial OD_600nm_ of 0.1, the temperature was set at 30 °C, and the pH was controlled at 6.7 by addition of NH_4_OH 30% (w/v). The dissolved oxygen concentration was kept higher than 30% by the controller software, which automatically changed the stirrer speed, varying between 200 and 1200 rpm, and also added pure oxygen to the air stream supplied to the bioreactor with a total gas flow rate at 6 L/min. Samples were collected hourly for determination of cell concentration by measurement of OD_600nm_. The supernatant of the samples was also collected and stored at − 20 °C for quantification of glucose and organic acids. At the point when the OD_600nm_ of the culture reached 10.0 (9 h cultivation), the inducer IPTG was added to a final concentration of 0.1 mM and followed for 3 h. After 12 h cultivation, the biomass was harvested from the culture broth by centrifugation, at 4 °C and 6693 × g, for 30 min. The recovered cell pellet was then transferred to plastic bags and stored at − 80 °C.

### Bioreactor culture evaluation

The carbon source consumption and production of organic acids were analyzed by HPLC using an Aminex HPX-87H column (300 cm × 7.8 mm; 9 µm particle size; 8% cross linkage) (Bio-Rad, USA) attached to 1260 Infinity (Agilent Technologies, USA) and using a system flow of 0.6 mL/min of a 5 mM sulfuric acid as the mobile phase at 60 °C. The refraction index detector was used for detection of sugars, and the UV detector at 210 nm for organic acids. Standard solutions with known concentration of glucose (1.0 to 5.0 g/L), oxalic acid (0.2 to 1.6 mM), citric acid (1.0 to 8.0 mM), malic acid (2.0 to 16 mM), formic acid (5.0 to 40 mM), and acetic acid (10 to 80 mM) were used for quantification of the components. For plasmid stability assessment, samples of the culture, collected right before induction and each hour after the induction, were diluted, plated on LB agar, and incubated for 24 h at 37 °C. Then, at least 100 colonies from each timepoint were simultaneously replicated in LB agar with and without kanamycin. After incubation at the same conditions, the percentage of antibiotic-resistant colonies in relation to the total colonies recovered without antibiotic was calculated and defined as the plasmid stability. The results obtained, together with the cell concentration measurement, were used to build the graph using the software Origin 2022b (OriginLab, USA).

### PspA1 purification protocols

**Fig. 1:**
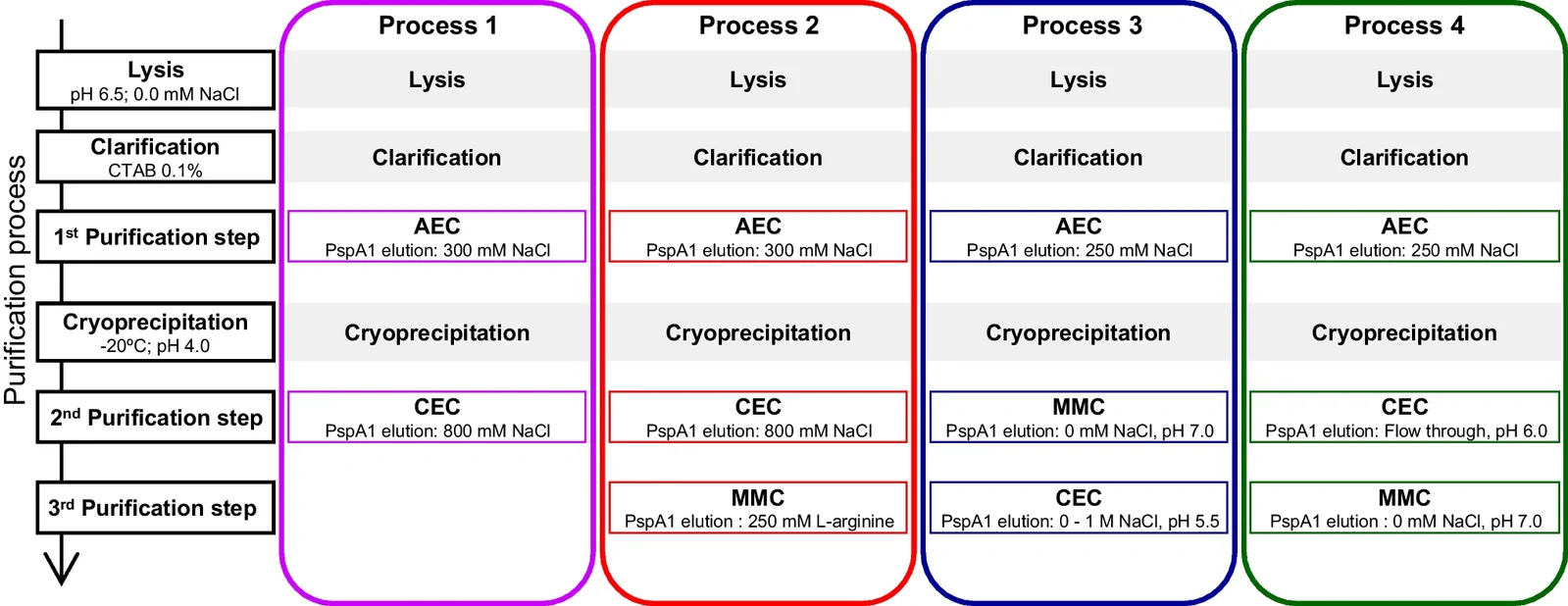
Scheme of purification processes tested to obtain a highly pure untagged recombinant PspA1. The different processes applied for the purification of the untagged recombinant PspA1 kept unchanged the conditions of lysis, clarification, and cryoprecipitation, but varying the conditions in the steps of AEC, CEC, and MMC

Three protocols for the purification process were tested before definition of the final purification process as shown in Fig. 1. Based on the work established by Figueiredo et al. (2017) for purification of PspA4Pro, we initially tested the identical protocol, which includes lysis, clarification with cetyltrimethylammonium bromide (CTAB), anion exchange chromatography (AEC), cryoprecipitation at pH 4.0, and cation exchange chromatography (CEC). The second protocol tested the inclusion of multimodal chromatography (MMC) as the last step. The third purification protocol tested inversion of the order of steps, applying the cryoprecipitate to MMC, and followed by CEC. The fourth and final purification process protocol was defined as lysis, clarification, AEC, cryoprecipitation, CEC, and MMC.

### Cell disruption and clarification

First, the frozen wet cell pellet was suspended in lysis buffer (10 mM sodium phosphate; 2.5 mM EDTA; 0.1% (w/v) Triton X-100; 1 mM phenylmethanesulfonyl fluoride (PMSF); at pH 6.5) at a proportion of 100 g of biomass to 1 L buffer. Then, a shovel mixer was used at 4 °C and 11,000 rpm to homogenate and dissolve any clumps in the sample. For lysis, the cell suspension was recirculated through the high-pressure continuous homogenizer system APV Gaulin GmbH (Manton-Gaulin Company, USA) or passed through PandaPLUS® 2000 (GEA, Germany). The temperature was adjusted to 4 °C and cell disruption was performed by circulating the suspension into the close loop for 6 min at 600 bar and 1 L/min in the APV Gaulin or passed through the system 6 times at pressure range of 1200–1400 bar in the PandaPLUS. One sample was collected after this step and the recovered fraction was identified as homogenate.

The detergent CTAB was added to the homogenate to improve the efficiency of debris removal. The detergent was added to a final concentration of 0.1% (w/v) at pH 6.5. The CTAB was left in contact with the homogenate for 1 h at room temperature and 100 rpm. The mixture was centrifuged at 17,696 × g and 4 °C for 90 min. One sample was collected from the supernatant containing PspA1 and identified as clarified fraction.

### Anion exchange chromatography (AEC)

The AEC was carried out using different volumes of Q-Sepharose Fast Flow (GE Healthcare/Cytiva, UK) resin packed in columns from different sizes attached to Äkta Avant 150 (GE Healthcare/Cytiva, UK). The volumetric flow for all purifications was set at 2.50 mL/min·cm^2^. The system was equilibrated by passing through five times the column volume (CV) of 10 mM sodium phosphate buffer, pH 6.5. The elution was performed using a discontinuous gradient of NaCl. In the first strategy, the concentrations used were 150, 300, and 1000 mM in 10 mM phosphate buffer, pH 6.5. In the second strategy, NaCl was applied at 80, 250, and 1000 mM in 10 mM phosphate buffer, pH 6.5. In both strategies, 5 CV was used in each step of the gradient.

### Cryoprecipitation

The fraction containing PspA1 eluted from the AEC column was recovered and the pH of the sample was reduced to 4.0 by adding glacial acetic acid to the sample (Sigma-Aldrich/Merk, Germany). After pH adjustment, the sample was frozen at − 20 °C for 24 h. After this period, the fraction was thawed and centrifuged at 17,696 × g and 4 °C for 60 min. The supernatant with the soluble protein was recovered and identified as cryoprecipitate, pH 4.0.

### Cation exchange chromatography (CEC)

The CEC was carried out using different volumes of SP-Sepharose Fast Flow (GE Healthcare/Cytiva, UK) resin packed in columns from different sizes attached to Äkta Avant 150. The volumetric flow for all purifications was set at 2.26 mL/min·cm^2^. Two strategies were tested in this purification step. In the first strategy, the system was equilibrated with 5 CV of 25 mM sodium acetate buffer with 300 mM NaCl, pH 4.0. The elution was performed by a discontinuous gradient of NaCl at 500, 800, and 1000 mM in 25 mM acetate buffer, pH 4.0. In the second strategy, the system was equilibrated with 5 CV of 50 mM sodium acetate buffer, pH 5.5 or 6.0. The pH and conductivity of the fraction recovered from the previous step was corrected to match both the pH and conductivity of the equilibration buffer. After loading all the sample, the elution was performed by a linear gradient from 0 to 1000 mM of NaCl. In both strategies, the volume of buffer used in each step of the elution was 5 CV for the discontinuous gradient and 20 CV for the linear gradient.

### Multimodal chromatography (MMC)

The MMC was carried out using different volumes of Capto™ MMC (GE Healthcare/Cytiva, UK) resin packed in columns from different sizes attached to Äkta Avant 150. The volumetric flow for all purifications was set at 2.26 mL/min·cm^2^. Once again, two strategies were tested in this purification step. In both strategies, the pH and conductivity of the fraction recovered from the previous purification step were corrected to match both the pH and conductivity of the equilibration buffer of the strategy. In the first strategy, the system was equilibrated with 5 CV of 50 mM sodium acetate buffer, pH 6.5. The elution was performed by an isocratic elution using 5 CV of 250 mM L-arginine in 50 mM acetate buffer, pH 6.5. In the second strategy, the system was equilibrated with 5 CV of 50 mM sodium acetate buffer, pH 5.0. The elution was performed by an isocratic elution using 5 CV of 50 mM bis–tris buffer, pH 7.0.

### Protein quantification and determination of PspA1 purity and recovery

Protein concentration was determined by the adapted Lowry methodology (Lowry et al. 1951) using the DC® Protein Assay (Bio-Rad Laboratories, USA). PspA1 purity was first determined by densitometry of the bands (Laemmli 1970). Each lane of 12% SDS-PAGE was loaded with samples containing 15 to 10 µg total protein from the lysis, clarification, and AEC, 10 to 5 µg of total protein from the CEC, and 5 to 1 µg total protein from the MMC after adding 2-mercaptoethanol and boiling at 95 °C for 5 min. The percentage of PspA1 band was calculated in relation to the sum of all other bands in the lane using GS-800 (Bio-Rad Laboratories, USA) or DS-5000 (Loccus, Brazil). PspA1 purity was also determined by HPLC using a TSK-GEL G3000PW_XL_ column (30 cm × 7.8 mm; 7 µm; 200 Å) (Tosoh Bioscience, Japan) attached to the system 1260 Infinity. Phosphate-buffered saline (PBS) was used as mobile phase in a volume flow of 0.5 mL/min and 25 °C. Aliquots of 25 µL of samples from each purification step in concentration up to 1 mg/mL of total protein were loaded and run for 30 min. PspA1 purity was calculated as the percentage of the area from the peak at 14 min retention time, corresponding to PspA1, in relation to the sum of all areas of the sample.

The purification factor (PF) and protein recovery (Rc) were calculated according to Eqs. (1) to (5).

1 $${PF}_{global}=\frac{{PspA1(\%)}_{n}}{{PspA1 (\%)}_{h}}$$

2 $${FP}_{per step}=\frac{{PspA1(\%)}_{n}}{{PspA1 (\%)}_{sb}}$$

where *PspA*1 (%) is the purity determined by densitometry of PspA1 band in the homogenate fraction (*h*), in the step immediately before (*sb*), and in the step of analysis (*n*).

3 $$PspA1 (g)=\frac{Prot \left(g\right)\times PspA1(\%)n}{100}$$

where *PspA*1(*g*) is the PspA1 total amount and *Prot* (*g*) is the total amount of protein determined by Lowry method.

4 $${Rc(\%)}_{global}=\frac{{PspA1(g)}_{n}\times 100}{{PspA1(g)}_{h}}$$

5 $${Rc(\%)}_{per step}=\frac{{PspA1(g)}_{n}\times 100}{{PspA1(g)}_{sb}}$$

where *PspA*1(*g*) is the total mass of PspA1 in the homogenate fraction (*h*), in the step immediately before (*sb*), and in the step of analysis (*n*).

Lipopolysaccharide (LPS) was measured as endotoxin units by the Limulus amebocyte lysate test (Bang 1956) using the Pyrogent™ 125 Plus kit (Lonza, Switzerland).

### PspA1 sequence alignment and physico-chemical determinations

The theoretical isoelectric point and predicted mass of PspA1 were determined with the Prot pi: Protein Tool. The alignment of the amino acid sequences from PspA1 and PspA4Pro was performed with EMBOSS Needle version 6.6.0 (EMBL-EBI, UK). The prediction of burial of amino acids was performed with the JPred4 (Drozdetskiy et al. 2015).

### Secondary structure and thermal stability of purified PspA1

The secondary structure and thermal stability of PspA1 at different temperatures were assessed by circular dichroism (CD) using the spectropolarimeter J-810-150S (Jasco Corporation, Japan). Samples from the highest purity of PspA1 were diluted 100 × in water to reduce the absorptive effect of the buffer. For the secondary structure, measurements were obtained from 183 to 260 nm, and the final CD spectra resulted from the mean of five measurements. Deconvolution was calculated based on the Dichroweb online database (Whitmore and Wallace 2004) with CDSSTR algorithm (Sreerama and Woody 2000). For the thermal stability, the sample was submitted to an increase of temperature of 1 °C/min, from 2 to 98 °C and cooled back from 98 to 2 °C, and the alterations in the secondary structure were assessed at 222 nm. The results obtained from the readings were used to build graphs with the software Origin 2022b (OriginLab, USA). For the thermal stability analysis, the results were fitted in a sigmoidal non-parametric model and the inflection point of the curves (Tm) was determined from the heating, or positive variation of temperature (ΔT +), and cooling, or negative variation of temperature (ΔT −), of the sample.

### PspA1 binding to human lactoferrin assay

The binding ability of PspA1 to human lactoferrin was analyzed to assure that the biological activity of the protein was preserved after purification. In this assay, samples of 2 µg PspA1 from the highest purity fractions were submitted to 12% SDS-PAGE. As controls, 2 µg of BSA, PspA1 with His-tag purified by nickel affinity chromatography, and PspA4Pro were also included. After electrophoresis the samples were transferred to a nitrocellulose membrane. After blocking the membrane with 5% (w/v) bovine serum albumin (BSA) in tris-buffered saline and 0.1% (v/v) Tween® 20 (TBST), samples were incubated with 4 µg/mL human lactoferrin (Sigma-Aldrich/Merk, Germany) labeled with biotin using the Biotin Labeling kit (Roche, Switzerland). The membrane was then incubated with a dilution of 1:100 of streptavidin conjugated to horseradish peroxidase (R&D Systems, USA). Detection was performed using ECL™ Prime (GE Healthcare/Cytiva, UK) and Amersham Imager 680 (GE Healthcare/Cytiva, UK).

## Results

### Obtention of the PspA1 producing clone

**Fig. 2:**
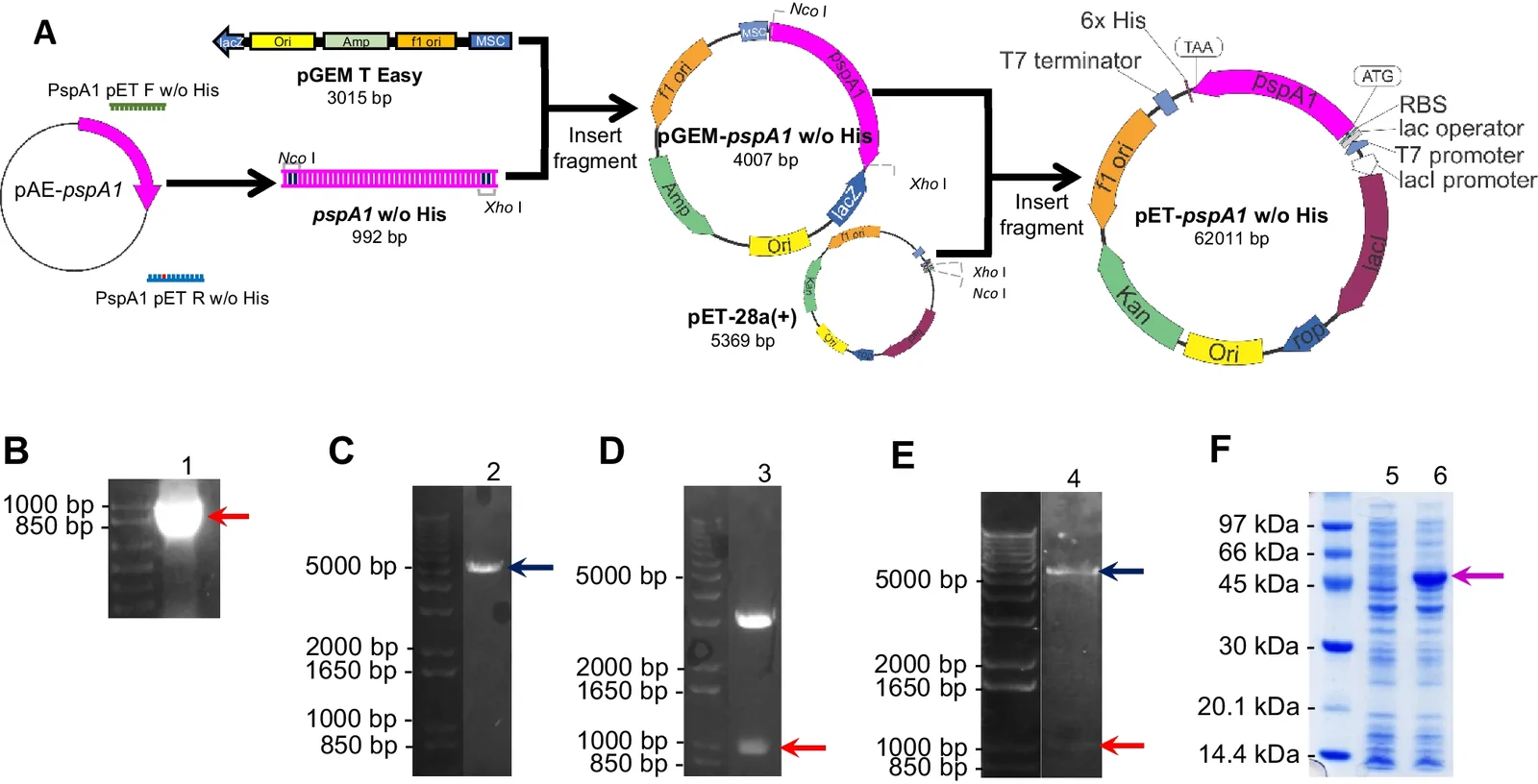
Cloning of *pspA1* and confirmation of production of an untagged recombinant PspA1 in *E. coli*. **A** Scheme showing the construction of the pET-*pspA1* plasmid. Plasmid pAE-*pspA1* was used as template for a PCR using primers containing *Nco* I and *Xho* I restriction sites and one stop codon at the 3′ end of *pspA1*. The amplified fragment was cloned into pGEM® T Easy, rendering pGEM-*pspA1* w/o His. pGEM-*pspA1* w/o His was then digested with *Nco* I and *Xho* I restriction enzymes for cloning of *pspA1* into pET-28a( +), digested with the same enzymes. **B** PCR amplification of *pspA1* (1). **C** Digestion of pET-28a( +) (2) with *Nco* I and *Xho* I restriction enzymes. **D** Digestion of pGEM-*pspA1* w/o His (3) with *Nco* I and *Xho* I restriction enzymes. **E** Final construct pET-*pspA1* w/o His after digestion with *Nco* I and *Xho* I (4) for confirmation of the insertion of *pspA1*. **F** *E. coli* BL21(DE3) transformed with pET-*pspA1* w/o His was cultivated until OD_600nm_ 0.6. At this point, a non-induced sample (5) was collected and the culture was induced with 0.1 mM IPTG. A sample was collected after 3-h induction (6) for SDS-PAGE analysis. Red arrows indicate the band of *pspA1*, dark blue arrows indicate the band of pET-28a( +), and purple arrow indicates the PspA1 protein

The construction of a plasmid for bacterial expression of *pspA*1 followed the steps depicted in Fig. 2A. Basically, plasmid pAE-*pspA*1 was used as template for the amplification of a 992-bp fragment encoding PspA1 as shown in Fig. 2B. After cloning into pGEM T Easy, the plasmid pGEM-*pspA*1 w/o His was sequenced for confirmation of the insert and then digested with restriction enzymes *Nco* I and *Xho* I. The digestion of pET-28a( +) was performed in parallel. The digestion of pET-28a( +) released a 5231-bp fragment (Fig. 2C), and pGEM-*pspA*1 w/o His released two fragments, pGEM T Easy (3015 bp) and *pspA*1 (992 bp) (Fig. 2D). The final construction was obtained by the ligation of *pspA1* to pET-28a( +), rendering pET-*pspA*1 w/o His. For confirmation of cloning, pET-*pspA*1 w/o His was digested with *Nco* I and *Xho* I, releasing two fragments of ~ 5000 and ~ 1000 bp (Fig. 2E). After confirming the construction pET-*pspA*1 w/o His, *E. coli* strain BL21(DE3) was transformed and cultivated in LB-Kan for analysis of gene expression. In Fig. 2F, PspA1 production is observed after 3-h induction with IPTG, represented by the band with high intensity above 45 kDa. The predicted molecular mass of the protein based on the amino acid sequence is 36.58 kDa (Fig. S1), but we have previously observed anomalous running of PspA in SDS-PAGE (Figueiredo et al. 2017). Using the equation proposed by Guan et al. (2015) for acidic peptides, the apparent molecular mass in SDS-PAGE for PspA1 would be 50.4 kDa.

### PspA1 producing clone adaptation to chemically defined medium and bioreactor culture

**Fig. 3:**
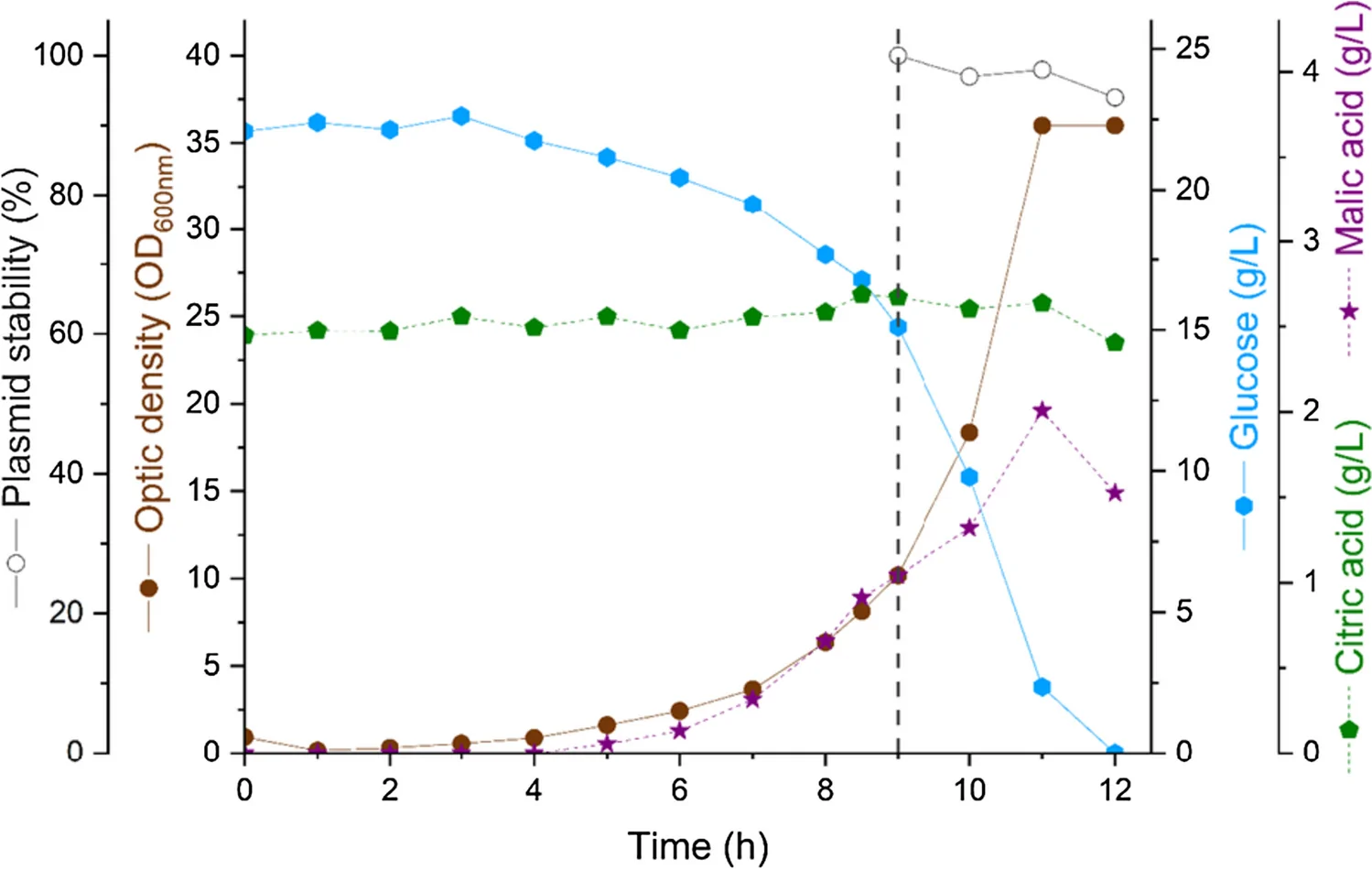
Cell growth profile, carbon source consumption, metabolite production, and plasmid stability in 10-L bioreactor cultivation for the production of untagged recombinant PspA1. *E. coli* BL21(DE3) transformed with pET-*pspA*1 w/o His was cultivated in a bioreactor and followed for 12 h. Samples were collected each hour to measure OD_600nm_, represented by brown dots and plotted in the vertical inner left axis. The supernatant of the samples was used for quantification of glucose, represented by blue hexagons and plotted in the vertical inner right axis; citric acid, represented by green pentagons, and malic acid, represented by purple stars, both plotted in the outer vertical right axis. The timepoint of induction is represented in the graph by the vertical dashed gray line. After this point, the plasmid stability was also determined, represented by the gray empty circles and plotted in the outer left vertical axis

**Fig. 4:**
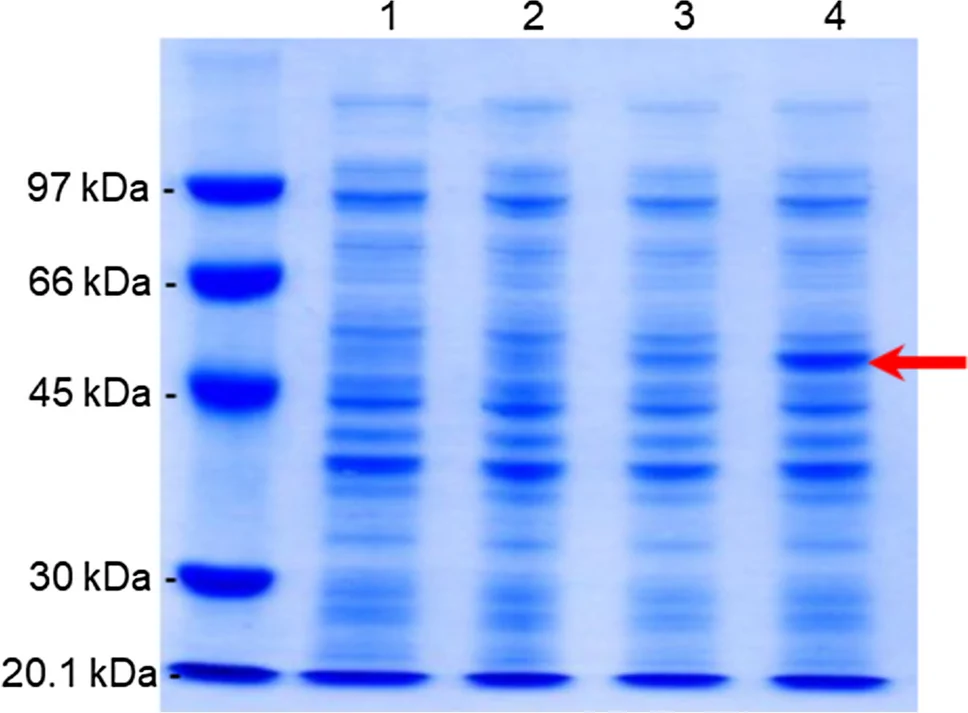
Analysis of the production of untagged recombinant PspA1 after 3-h induction in 10-L bioreactor. Samples recovered from the bioreactor right before the induction of the culture (1) and each hour after adding 0.1 mM IPTG (2–4) were diluted in buffer with SDS and 2-mercaptoethanol to obtain a OD_600nm_ of 10.0 for each sample and 10 µL loaded to each lane. The red arrow indicates PspA1 protein

The production of the seed stock of the *E. coli* strain BL21(DE3) producing PspA1 was based on selecting one clone on M9-Kan plate and cultivating it in M9-Kan broth. The seed stock was stored at − 80 °C in aliquots for single use. The initial cultivation in flasks using HDF medium, 16 h before the bioreactor cultivation, produced the cell quantity for bioreactor inoculation. With an initial OD_600nm_ of 0.1, progression of the culture was monitored and is shown in Fig. 3 as the brown dot series. The bioreactor culture needed 9 h to reach the induction point of OD_600nm_ equal to 10, when 0.1 mM IPTG was added (represented in Fig. 3 by the dashed gray line). The cell growth, measured by the OD_600nm_, stabilized at 2 h after induction. The plasmid showed high stability with more than 90% of resistant colonies in all timepoints after induction, represented in Fig. 3 as the empty gray circles. During the bioreactor culture, glucose concentration (blue hexagons in Fig. 3) was 22 g/L at the time of induction and dropped from 15 to 0 g/L after 1- and 3-h induction, respectively. The bioreactor culture showed no accumulation of acetic acid, which was not detected. Citric acid concentration (green pentagons in Fig. 3) showed some stability with concentrations ranging around 2.0 g/L, and malic acid concentration (purple stars in Fig. 3) reached a peak of 1.9 g/L at 11 h of culture, 2 h after induction, and started to decrease after that. The production of formic and oxalic acids (not shown) was negligible with concentrations around 0.2 g/L. Protein expression was confirmed by SDS-PAGE, shown in Fig. 4, with increasing intensity of the band of PspA1 after induction, that became evident at the end of the third hour of induction. The total time spent for the cultivation was 12 h and the weight of biomass recovered was 317 g.

### Purification processes tested for obtention of a highly pure PspA1

**Fig. 5:**
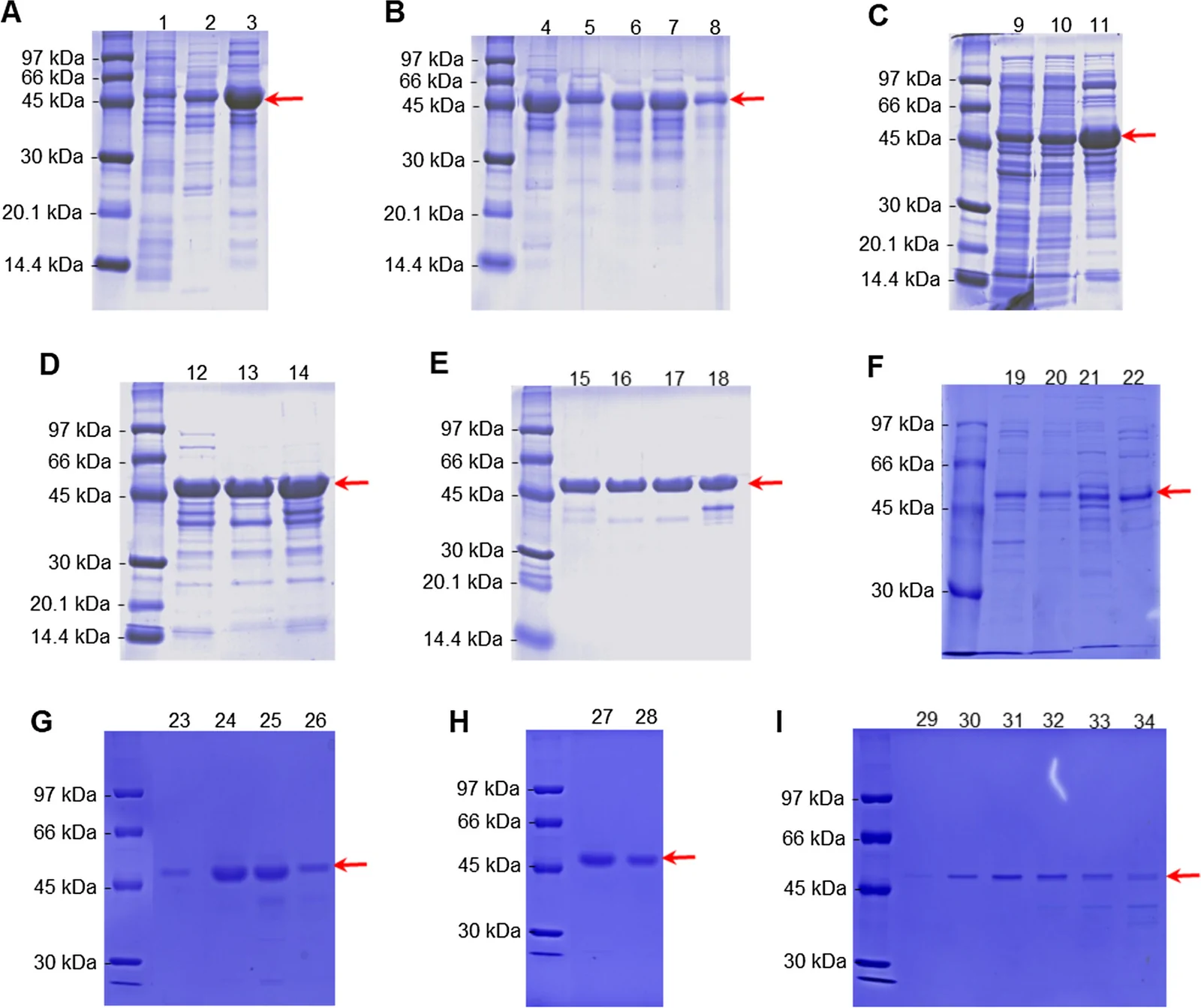
Analysis of the purity of the untagged recombinant PspA1 through the purification steps from three different processes tested. **A**, **B** The first process included steps of lysis (1), clarification with CTAB (2), AEC with elution using three different concentrations of NaCl (150 mM, 300 mM, and 1 M), and recovery of PpsA1 at 300 mM NaCl (3). The following steps included cryoprecipitation at pH 4.0 (4) and CEC with elution using concentrations of NaCl of 600 mM, 800 mM, and 1 M, and recovery of the PspA1 at 800 mM NaCl (5–8). **C**–**E** The second process kept the same conditions for lysis (9), clarification with CTAB (10), AEC (11), cryoprecipitation (12), and CEC (13–14). The inclusion of MMC with elution of PspA1 using L-arginine showed recovery of the protein in the flowthrough (15), after the column wash (16), and in the 250 mM L-arginine elution fractions (17–18). **F**–**I** The third process tested kept unaltered the steps of lysis (19) and clarification with CTAB (20). For AEC, the elution was performed with 80 mM, 250 mM, and 1 M NaCl, and PspA1 was eluted at 250 mM NaCl (21–22). The step of cryoprecipitation (23) was not altered, but the following step was changed for MMC using pH 5.0 to equilibrate the column, followed by increasing it to 7.0 without addition of NaCl for elution (24–26). For the next step, the fractions recovered from the MMC were applied to a CEC column and the elution was done using a linear gradient of NaCl from 0 to 1 M at pH 5.5. PspA1 was recovered in the flowthrough (27–28) and in the gradient of NaCl (29–34). The protein content in each sample was quantified by the Lowry colorimetric method. For samples recovered after lysis, clarification, and AEC, 15 µg (**A** and **C**) or 10 µg (**F**) of total protein was loaded per lane. For samples from the cryoprecipitation, 5 µg (**B** and **G**) or 10 µg (**D**) of total protein was loaded per lane. For samples from CEC, 5 µg (**B**, **H**, and **I**) or 10 µg (**D**) of total protein was loaded per lane. For MMC samples, 5 µg (**E** and **G**) of total protein was loaded per lane. The red arrows indicate PspA1 protein

For the definition of the purification process, three different processes were tested. In the first purification process tested, despite the differences between PspA1 and PspA4Pro in the amino acid sequence (Fig. S2 and Fig. S3), we tried to replicate the process of purification established before by Figueiredo et al. (2017). For that reason, 10 g of biomass obtained from the bioreactor culture, stored at − 80 °C, was suspended in lysis buffer at pH 6.5 and passed through the high-pressure homogenizer (Fig. 5A). The homogenate was then treated with CTAB for obtention of the clarified fraction (Fig. 5A). After centrifugation, the sample was applied to the AEC column; a discontinuous gradient of 150, 300, and 1000 mM NaCl was applied; and PspA1 was recovered in the fraction with 300 mM NaCl (Fig. 5A). This fraction was then submitted to the cryoprecipitation step, in which PspA1 was recovered in the supernatant (Fig. 5B). As the final step of the process, the cryoprecipitate was applied to the CEC column for recovery of PspA1 in the fraction with 800 mM NaCl, after a discontinuous gradient of 600, 800, and 1000 mM NaCl (Fig. 5B). At the end of this process, it is possible to see in Fig. 5B bands above and below the PspA1 band in the final fraction, resulting in PspA1 purity below 90%.

The second purification process tested included a lysis of 80 g of biomass, following the same proportion of biomass-to-lysis buffer established before (Fig. 5C). The steps of clarification with CTAB and recovery of PspA1 at 300 mM NaCl after AEC were kept unaltered (Fig. 5C). The cryoprecipitation step and the recovery of the PspA1 at 800 mM NaCl after CEC was also kept unaltered (Fig. 5D). The inclusion of the MMC for recovery of PspA1 by elution with 250 mM L-arginine showed low recovery, since the presence of PspA1 was observed in the flowthrough and column wash, right before the elution with L-arginine. We also observed the presence of bands with molecular weight below PspA1 after elution with L-arginine, which reduced the final purity (Fig. 5E).

The third process tested kept unaltered the steps of lysis and clarification with CTAB and used 20 g of biomass produced in the bioreactor culture (Fig. 5F). For the AEC, the elution was performed by a discontinuous gradient of 80, 250, and 1000 mM NaCl, and PspA1 was recovered in the 250 mM NaCl fraction (Fig. 5F). The cryoprecipitation step was not altered (Fig. 5G). In this process, the order of the final chromatography steps was inverted, and the elution strategy was changed. The supernatant of the cryoprecipitation was submitted to MMC after reducing the conductivity and correcting the pH of the sample to 5.0. PspA1 was then eluted with the buffer at pH 7.0 (Fig. 5G). The final step of the process was performed by a CEC applying a linear gradient from 0 to 1 M NaCl after correcting the pH of the sample to 5.5. When loaded at pH 5.5 into CEC, PspA1 was obtained in the flowthrough (Fig. 5H) with less contaminant bands than during the gradient elution, and elution fractions retained most contaminants (Fig. 5I).

### Obtention and characterization of the highly pure PspA1

**Fig. 6:**
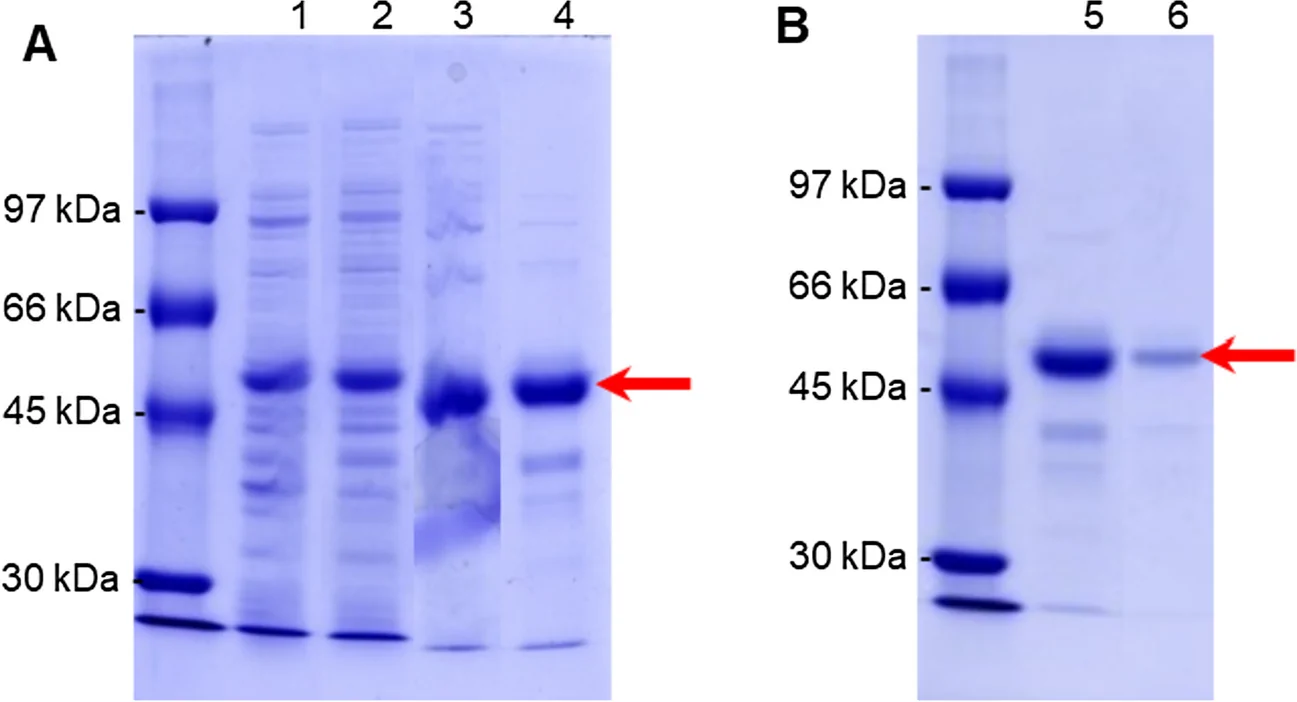
Analysis of the purity of the untagged recombinant PspA1 through the steps of the final purification process. For the final process, the steps with the highest purity of PspA1 were selected. **A** The lysis (1) and clarification with CTAB (2) were kept unaltered. For AEC, the conditions were similar to the process 3, with elution of PspA1 with 250 mM NaCl (3). The cryoprecipitation step was also kept unaltered (4). **B** The fraction recovered from the cryoprecipitation was applied to the CEC column for recovery of the protein in the flowthrough using pH 6.0 (5) and followed by a MMC step and elution of PspA1 at pH 7.0 without NaCl (6). The protein content in each sample was quantified by the Lowry colorimetric method. The samples were loaded in the gel by using a total amount of protein in each lane of 10 µg (1–3), 5 µg (4 and 5), or 1 µg (6). The red arrows indicate the PspA1 protein

For the final purification process for a highly pure PspA1, the purification steps were chosen based on maximum recovery and purity of the protein obtained from each protocol tested. The purification process started with the lysis of 80 g biomass suspension, using the high-pressure homogenizer (Fig. 6). The homogenate was treated with CTAB and loss of PspA1 in the insoluble fraction is negligible or not existent (Fig. S4). Then, the clarified fraction was applied to the AEC column, in which PspA1 was recovered with 250 mM NaCl. This fraction was used for the cryoprecipitation at pH 4.0 and PspA1 was recovered in the soluble fraction, again the loss of PspA1 in the insoluble fraction is negligible or not existent (Fig. S4). The supernatant of the cryoprecipitation step was then applied to the CEC column after reducing the conductivity of the sample by dilution with ultra-pure water and correcting the pH to 6.0. PspA1 was recovered in the flowthrough fraction of the CEC, which was applied to the MMC column after correcting the pH of the sample to 5.0. Finally, PspA1 with the highest purity was recovered after elution with bis–tris buffer at pH 7.0. In Fig. 6, it is possible to see the presence of a ~ 50-kDa band, corresponding to PspA1, in all fractions and the reduction of contaminant bands during the process. At the end of the process, only a faint band below the ~ 50-kDa band of PspA1 was visible.

Based on the analysis of the SDS-PAGE by densitometry combined with the protein quantification from each step, calculations were made to determine the efficiency of the process. The results from the final purification process were summarized in Table 1. When using a 14 mL MMC column at the end of the process, the final recovery of PspA1 was 21.0% and recovery from the previous step was 62.3%. In order to increase the final recovery of PspA1, the process was repeated but using a 58 mL MMC column, which increased the final PspA1 recovery to 28.6% and recovery from the previous step to 84.8%. With this process, it was possible to recover a protein amount of 644 mg with purity of 84% with 14 mL MMC column or 877 mg with 86% purity with the 58 mL MMC column. The amount of protein recovered after changing the volume of the MMC column represented an increase of 36% of protein. Among all the purification steps, AEC followed by cryoprecipitation and clarification were the most important steps to increase the purity of the protein, which showed purification factors per step of 1.7, 1.6, and 1.1, respectively. However, the final purity of the protein analyzed by densitometry was less than 90%, despite only a faint contamination band being observed in SDS-PAGE (Fig. 6 lane 6). For confirmation of the protein purity, samples using the same purification protocol were reanalyzed in parallel by densitometry and HPLC. As seen in Table 2, it was possible to characterize the final fraction of PspA1 with purity of 93% using HPLC, whereas the same fraction showed a purity of 86% when analyzed by densitometry.

**Table 1:** Determination of the purity and recovery of the untagged recombinant PspA1 using the established final process

Purification steps	PspA1 purity (%)	PspA1 mass (g)	PspA1 Rc (%) (global)	PspA1 Rc (%) (per step)	PF (global)	PF (per step)
Homogenate	27	3.1	100	100	1.0	1.0
Clarified fraction	30	2.6	84.0	84.0	1.1	1.1
Anion exchange chromatography						
250 mM NaCl, pH 6.5	50	1.7	56.8	67.6	1.9	1.7
Cryoprecipitate, pH 4.0	79	1.2	38.8	68.3	3.0	1.6
Cation exchange chromatography						
0 mM NaCl, pH 6.0—flowthrough	83	1.0	33.7	86.9	3.1	1.1
Multimodal chromatography 0 mM NaCl, pH 7.0						
1st process—column volume: 14 mL	84	0.64	21.0	62.3	3.1	1.0
2nd process—column volume: 58 mL	86	0.88	28.6	84.8	3.2	1.0

*Rc*, recovery; *PF*, purification factor

**Table 2:** Comparison of the purity of the untagged recombinant PspA1 in the final process by densitometry and HPLC methods

Purification steps	PspA1 purity (%) Densitometry	PspA1 purity (%) HPLC
Homogenate	10	4.8
Clarified fraction	14	11
Anion exchange chromatography	33	21
Cryoprecipitate	43	44
Cation exchange chromatography	51	50
Multimodal chromatography	86	93

This final purification process was performed under conditions to reduce endotoxin contamination from the environment, thus the materials used were sterilized and depyrogenated, as well as the water used for cleaning chromatography columns and preparing buffers had low endotoxin content. For confirmation of the endotoxin content at the end of the process, a sample from the MMC was collected for the measurement. The final PspA1 fraction with a concentration of 10 mg/mL had less than 0.5 EU/mL.

**Fig. 7:**
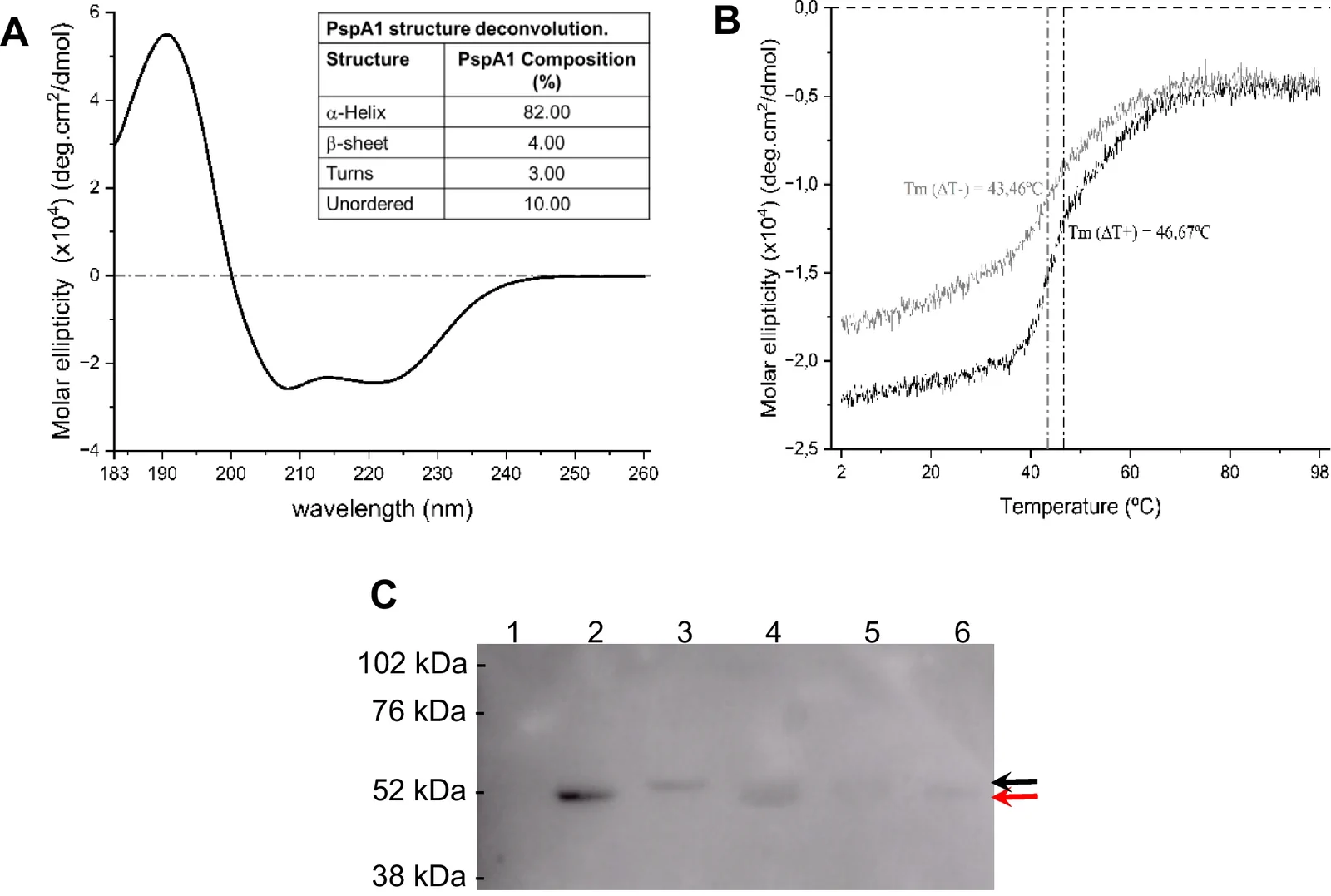
Characterization of the structure, thermal stability, and biological activity of the untagged recombinant PspA1. **A** Sample of the highly pure protein, recovered from the final purification process, was diluted in water for circular dichroism analysis using a wavelength range varying from 183 to 260 nm. The results obtained were used for deconvolution and determination of the structure of the protein (inset table). **B** The thermal stability was also assessed using a fixed wavelength at 222 nm and submitting the sample to a variation of temperature from 2 to 98 °C (black) or from 98 to 2 °C (gray). The dashed lines represent the temperature when the inflection point of the sigmoidal curve of the sample during heating (Tm(ΔT +)) (black) and cooling (Tm(ΔT −)) (gray) occurred. **C** The biological activity was determined by evaluation of binding of lactoferrin to PspA1. As controls, 2 µg of BSA (1), PspA1 with His-tag purified by nickel affinity chromatography (2), and PspA4Pro (3) were used. Fractions from purifications (4–6) using the final protocol were also added (2 µg of total protein). After transfer to nitrocellulose membrane and blocking, samples were incubated with 4 µg/mL biotinylated lactoferrin followed by streptavidin–horseradish peroxidase conjugate. The red arrow indicates PspA1 and the black arrow indicates PspA4Pro

The protein obtained from the final process was then characterized by its secondary structure and biological activity. The secondary structure was assessed by CD using a range of wavelength from 183 to 260 nm. This result is presented in Fig. 7A, and in the inset of Fig. 7A, the result of deconvolution of the PspA1 spectrum is presented. It was possible to observe that PspA1 exhibited 82% of its structure organized as α-helix, 4% as β-sheet, 3% as turns, and 10% as unordered structure. The thermal stability of the PspA1 was also assessed and is shown in Fig. 7B. PspA1 showed only one point of inflection in the sigmoidal curve after fitting in the model, which represents the melting temperature (Tm). Also, it seems that PspA1 lost all the structure above 60 °C and did not recover it completely after cooling down, resulting in different Tm during heating and cooling (Fig. 7B).

The biological activity was characterized by evaluating the binding of the protein to human lactoferrin conjugated to streptavidin. As seen in Fig. 7C, samples from the final step from the final purification process were separated by SDS-PAGE and then transferred to a nitrocellulose membrane. BSA, PspA1 from purification by nickel affinity chromatography, and PspA4Pro were included as controls. After incubations, it was possible to see that the protein retained its ability to bind to human lactoferrin, indicating the preservation of the epitope responsible for this interaction in the protein recovered from the process.

## Discussion

PspA is an important antigen for the development of an alternative vaccine against pneumococcal disease. Because of its variability, it is necessary to include a PspA from family 1 and a PspA from family 2 in vaccine formulations in order to induce broader response. We formerly described the production and purification of an untagged recombinant PspA4Pro (clade 4, family 2) (Figueiredo et al. 2017). In this work, we have demonstrated and characterized the process for obtaining recombinant PspA1 (clade 1, family 1) with purity above 90% as a new antigen for inclusion in vaccines against pneumococcal infections.

The PspA1 gene sequence encoding the mature N-terminal and the proline-rich domains was cloned into pET-28a( +). For obtaining a PspA1 expressing clone, the commercial strain BL21(DE3) was chosen based on the fact that this strain has been established for protein production, producing 8 times more protein than the wild strain. This was obtained after the inclusion of the bacteriophage T7 RNA polymerase into the genome of this strain, which increased the gene expression for transcription of almost every DNA linked to a T7 promoter (Jeong et al. 2009; Studier and Moffatt 1986).

The adaptation of the PspA1 producing clone to HDF medium was an important step to establish a process without components of animal origin, minimizing the risk of development of spongiform encephalopathy from the final product, required by the WHO (WHO - World Health Organization 2009). The chemically defined medium, HDF, previously established by Riesenberg et al. (1991) has been used for years in high cell density cultures. It was designed for maximizing cell growth, while minimizing formation of fermentation by-products. Other important factor is that using chemically defined media, such as HDF, for protein production in bacterial cultures can reduce the costs of the final product, since the price of the medium represents 80% of all costs spent for a single batch (Cardoso et al. 2020). For the induction of PspA1 gene expression, IPTG was selected based on a previous comparison with lactose, which showed IPTG as a more efficient inducer for PspA production (Horta et al. 2012). For PspA production in *E. coli* specifically, it has been described that short-term culture with temperature around 32 °C and induction with IPTG is the best cost-effective production method (Cardoso et al. 2020). As expected for HDF medium, the production of the inhibitory component acetic acid was not detected in the bioreactor culture, and other organic acids, such as citric and malic acids, formed in the tricarboxylic acid (TCA) cycle as part of cell metabolism, were detected below inhibitory concentration values. The transformed strain showed a plasmid stability > 90% even after 3-h induction, enabling a production of 2.1 g/L of PspA1. The intense cell production, showed by the rapid increase of OD_600nm_, resulting in high cell concentration in the bioreactor correlates with the drastically reduction of the glucose content in the medium after the induction. This high yield is in agreement with results obtained by Figueiredo et al. (2017) and Carvalho et al. (2012) with production of different PspAs in bioreactor.

For the establishment of the conditions for the purification process, the steps were chosen based on the definitions from WHO - World Health Organization (2009), which requires that the proteins to be included in PCVs, as carriers for PS, must have more than 90% of purity. This parameter was used because there is no protein vaccine licensed against pneumococcus yet. The first purification process tested was a simple reproduction of the method described by Figueiredo et al. (2017), which included lysis at pH 6.5, clarification with CTAB, AEC with elution of PspA in 300 mM NaCl, cryoprecipitation at pH 4.0, and CEC with elution of PspA4Pro in 800 mM NaCl. It was clear that this process was not efficient to achieve the same purity of PspA1 as obtained for PspA4Pro. This result might be explained by a combination of factors. First, the differences between the amino acid sequences of the two proteins prompted alterations in the residues exposed on the surface of PspA1 for interaction with the resins. PspA1, for example, has higher percentage, except for the hydrophilic residues, of predicted burial of amino acids than PspA4Pro, which corroborates with the poor interaction with the resins. Another factor is the lack of some residues or moieties in the PspA1 sequence that may be responsible for the interactions of PspA4Pro with the resins. The comparison of the amino acid composition of both proteins showed they are quite similar (PspA1: 39.71% hydrophobic residues, 14.16% hydrophilic residues, 26.77% acidic residues, and 19.39% basic residues; PspA4Pro: 38.29% hydrophobic residues, 16.8% hydrophilic residues, 25.62% acidic residues, and 19.28% basic residues), but PspA4Pro has a larger sequence which leads more residues to interact with the resins. Also, the amino acids present only in the PspA4Pro are mostly hydrophobic (42.14%), followed by acidic (24.79%), hydrophilic (19.01%), and basic (14.05%). These factors combined led to modifications in all the chromatographic steps of the purification process.

The second purification process tested included a MMC after the AEC and employed L-arginine for the elution of the protein from MMC. This step showed some improvement with a fraction showing PspA1 purity of 94%, but low recovery due to the loss of the protein during the loading and column wash, which made us hypothesize that this protein weakly adsorbed to the resin in a way that NaCl could remove it. This was quite the opposite of PspA4Pro, which adsorbed so strongly to the multimodal resin that it was not eluted even with 1 M NaCl (Figueiredo et al. 2017). The weak interaction of PspA1 may be explained by the lower percentage of exposed residues, especially the hydrophobic residues. This unique behavior of PspA1 led us to alter the process by reducing the conductivity of the sample before loading to the MMC column and using pH change as strategy for elution of the protein. In fact, Holstein et al. (2012) had shown that in order to increase resolution of the separation of a mixture of proteins using MMC, the pH alteration was a better solution than using NaCl gradient. Arakawa et al. (2015) also showed that the concentration of NaCl can increase the strength of the hydrophobic interaction by salting-out effect, indicating the necessity to combine pH change and salt concentration for elution of protein strongly bound to the MMC resin. Since PspA1 has more buried hydrophobic residues, this could explain why this protein poorly adsorbed to MMC resin, indicating the necessity of alterations in this process.

For the third purification process tested, the concentration of NaCl used for the elution steps in the AEC was reduced and the order of the final steps was altered. So, the soluble fraction recovered from the cryoprecipitation was submitted to MMC after reducing NaCl concentration from 250 to 150 mM and elution of PspA1 was achieved at pH 7.0. Then, the MMC fraction was applied to the CEC column after reducing the pH to 5.5 and the elution of PspA1 was performed with a linear gradient from 0 to 1 M NaCl. MMC using pH elution showed some increase in the purification factor, but it was not efficient to remove all the contaminants. In addition, CEC was more efficient in retaining the contaminants adsorbed to the resin and released a highly pure PspA when using pH above the theoretical pI (4.79) of the protein, and when the gradient started, the contaminants were eluted together with PspA1. In fact, some works have been focusing on the development of processes based on flowthrough polish, also called negative chromatography. Weigel et al. (2014), for example, demonstrated an integrated flowthrough recovery intercalating different resin types to purify influenza virus, which resulted in high yield with low content of DNA. This type of purification process has also been applied to other pharmaceutical drugs, such as monoclonal antibodies (Ichihara et al. 2019, 2018; Yamada et al. 2017).

For the final purification process, the best conditions for PspA1 purification were chosen. The first steps were kept unaltered from the last process and comprised the lysis, clarification, elution with 250 mM NaCl in the AEC, and cryoprecipitation. The order of resins from the final steps were changed again, because negative CEC increases sample volume and MMC reduces it, making it advantageous to left the MMC to the end. Thus, the soluble fraction recovered from the cryoprecipitation was applied to the CEC column after increasing the pH to 6.0, which is important to decrease the protonation of the amino acids and turned the protein more negatively charged on the surface, thus PspA1 did not adsorb to the resin and was recovered in the flowthrough. The sample was diluted to reduce NaCl concentration, which could remove the contaminants adsorbed onto the resin during the loading of the sample into the CEC column. Thus, the flowthrough of CEC was applied to the MMC, which acted both as a polishing method removing the residual contaminants and as a step to concentrate the final protein.

This strategy showed visible efficiency in the final purity of the PspA1 analyzed by SDS-PAGE. After analyzing each step by densitometry, it was possible to determine that the process presented an overall purification factor of 3.1 or 3.2 when a 14 or 58 mL MMC resin, respectively. The recovery of the protein was also increased by augmenting the MMC column volume. But again, in both processes with different column volumes, the final purity determined by densitometry was below 90%. We evaluated a HPLC size exclusion method for assessment of the purity of the protein, which showed a final PspA1 purity of 93%. Even though the purity described here was below the 97.8% purity of PspA4Pro described by Figueiredo et al. (2017), the process described here has some advantages. AEC had more impact in the purity increase, with a purification factor of 1.7, than the clarification, with purification factor of 1.1. These results are different from what was shown in the PspA4Pro purification process, in which AEC and clarification showed purification factor of 1.3 and 1.6, respectively. On the other hand, the overall purification factor increased from 2.96 for the PspA4Pro process compared to 3.1 (14 mL MMC) or 3.2 (58 mL MMC) using densitometry or 19 (58 mL MMC) using HPLC for the method described here. In his work, Figueiredo et al. (2017) also showed that the clarification was the most important step to remove endotoxins and by the end of the process, endotoxin content was 0.07 EU/mg PspA4Pro. We demonstrated that the process described here maintained low endotoxin content by the end of the process with less than 0.05 EU/mg PspA1. For comparison, the recommendation is that endotoxin content for recombinant and polysaccharide vaccines should be below 20 EU/mL. In the 7-valent PCV, endotoxin content in a single dose of 500 µL was 0.21 EU (Brito and Singh 2011). In 2000, Briles et al. (2000) showed in a clinical trial that immunizing healthy humans with 125 µg of PspA from clade 2 twice induced serum antibodies that passively protected mice against pneumococcal infection. For this dose, the amount of endotoxin in PspA1 would be less than 0.006 EU/dose and one single purification process could produce more than 7000 doses.

The characterization of the final protein recovered showed that the majority of PspA1 was structured as α-helix with 82%, followed by 10% of unordered sequence, 4% β-sheet, and 3% turns, as expected. For PspA4Pro, the protein showed the same percentage of α-helix, but there were differences in unordered sequence (4%), turns (6%), and β-sheet (8%) (Figueiredo et al. 2017). PspA1 also showed only one Tm point for both heating and cooling processes, whereas PspA4Pro had shown 2 Tm points for each, which means that two points of inflection in the sigmoidal curve could be fitted for PspA4Pro during heating and other two when it was cooled back. This indicates that PspA4Pro first lost part of its structure, resist for some increase of temperature, and then finally lost all its structure, while PspA1 lost all its structure at once when the temperature is above 60 °C. It was also possible to observe that PspA1 showed different molar ellipticity curves at 222 nm in the range of 2 to 40 °C during heating and cooling, which indicates that PspA1 could not recover completely its secondary structure. On the opposite, when PspA4Pro was cooled back, it recovered the original secondary structure, which indicates that PspA4Pro is more thermoresistant than PspA1.

In conclusion, we showed here an easily scaled-up process for production and purification of a PspA from clade 1. The difference between PspA4Pro and PspA1 in the amino acid sequence of the molecules imposed the development of a novel method for purification. The final protein showed a purity above 93% when analyzed by size exclusion HPLC. The final protein showed reduced endotoxin content and preserved its secondary structure and biological activity.

## Supplementary Information

Below is the link to the electronic supplementary material.

Supplementary file1 (PDF 438 KB)

## Data Availability

All data generated or analyzed during this study are included in this published article and in the supplementary file. Please turn to the corresponding author for all other requests.
