# Supplementary material for: Novel method for production and purification of untagged pneumococcal surface protein A from clade 1
Source: Appl Microbiol Biotechnol. 2024 Apr 4;108(1):281. doi: 10.1007/s00253-024-13098-2 (PMC10990985; doi:10.1007/s00253-024-13098-2)
Supplement: Supplementary file 1 — Supplementary file1 (PDF 438 KB) [file 253_2024_13098_MOESM1_ESM.pdf]

**Applied Microbiology and Biotechnolgy**

**Supplementary material**

# NOVEL METHOD FOR PRODUCTION AND PURIFICATION OF UNTAGGED PNEUMOCOCCAL SURFACE PROTEIN A FROM CLADE 1

Tasson da Costa Rodrigues [1,2], Patricia Zorzete [3], Eliane Namie Miyaji [1,2], Viviane Maimoni Gonçalves [2,3,*].

1 - Laboratório de Bacteriologia, Instituto Butantan, São Paulo (São Paulo), Brazil;

2 - Programa de Pós-Graduação Interunidades em Biotecnologia, Universidade de São Paulo, São Paulo (São Paulo), Brazil;

3 - Laboratório de Desenvolvimento de Vacinas, Instituto Butantan, São Paulo (São Paulo), Brazil;

* Corresponding author: Viviane Maimoni Goncalves

Av. Vital Brasil, 1500

05503-900 São Paulo SP

Brazil

e-mail: viviane.goncalves@butantan.gov.br

phone: +55 11 26279819

## A

TAG**CC**ATGGAAGAAGCGCCCGTAGCTAGTCAGTCTAAAGCTGAGAAAGACTATGATGCAGCAGTGAAAAAATATGAAGCT
GCTAAGAAGGAATATGAGGACGGAAAAGCTGCTCAGAAAAAGTATGAAGATGATCAGAAGAAAACTGAGGAGAAAGCGGA
AGAAGAAAGAAAAGCTTCTGAAGAAGAACAAGCTGCAAATCTGAAATATCAACAAGAGTTGGTTAAATATATACGTGAAA
ATGATCCAACAAAAAAAGCTGAAGCTAAGAAAGCAATGGATGAGGCTGAGAAAGAGTATAAGAAAAAACAAACGGAATTT
GCTGAAGTTCGTGCAAAGGTAATTCCTAGCGCGGAAGAATTAAAAAAGACTAGACAAAAAGCAGAAGAGGCTAAAGCAAA
AGAAGCAGAACTTACTAAAAAAGTAGAAGAAGCTGAGAAAAAAGTTACTGAAGCCAAACAAAAAGTGGATGCAGAACATG
CTGAAGAAGTCGCTCCTCAAGCTAAAATCGCTGAATTGGAACATGAAGTTCAGAAACTAGAAAAAGCTCTCAAAGAGATT
GATGAGTCTGATTCAGAAGATTATGTTAAAGAAGGTCTCCGTGCTCCTCTTCAATTTGAATTGGATGTTAAGCAAGCTAA
ACTATCAAAACTTGAAGAGTTGAGTGATAAGATTGATGAGTTAGACGCTGAAATTGCAAAACTTGAAAAAGATGTAGAAG
ATTTCAAAAACTCAGACGGTGAGCAAGCTGGACAATACCTTGCTGCAGCTGAAGAAGACTTAGTTGCTAAAAAAGCTGAA
TTAGAAAAAACTGAAGCTGACCTTAAGAAAGCAGTTAATGAGCCAGAAAAACCAGCTGAAGAAACTCCAGCTCCAGCACC
AAAACCAGAGCAACCAGCTGAACAACCAAAACCAGCGCCGGCTCCTCAACCAGAAAAACCAGCTGAAGAGCCTGAGAATC
CAGCTTCAGCACCACAACCATAA**CTCGAG**CTA

## B

MEEAPVASQSKAEKDYDAAVKKYEAAKKEYEDGKAAQKKYEDDQKKTEEKAEEERKASEEEQAANLKYQQELVKYIREND
PTKKAEAKKAMDEAEKEYKKKQTEFAEVRAKVIPSAEELKKTRQKAEEAKAKEAELTKKVEEAEKKVTEAKQKVDAEHAE
EVAPQAKIAELEHEVQKLEKALKEIDESDSEDYVKEGLRAPLQFELDVKQAKLSKLEELSDKIDELDAEIAKLEKDVEDF
KNSDGEQAGQYLAAAEEDLVAKKAELEKTEADLKKAVNEPEKPAEETPAPAPKPEQPAEQPKPAPAPQPEKPAEEPENPA
SAPQP

Theoretical isoelectric point (pI): 4.79

Theoretical molecular weight: 36579.62 Da

**Fig. S1** DNA and amino acid sequence of the untagged recombinant PspA1. A - The insert of 992 bp of *pspA1*, obtained after the PCR and confirmed by sequencing. The sequence comprises the N-terminal and proline rich domains of PspA1. The highlighted sequences indicate the region with homology with PspA1 pET F w/o His (light gray) with *Nco* I restriction site (bold) and start codon (green), and PspA1 pET R w/o His (dark gray) with *Xho* I restriction site (bold) and stop codon (red). B - The mature amino acid sequence of the PspA1 was produced in BL21 (DE3) strain using the pET-*pspA1*. The theoretical isoelectric point and molecular weight were calculated based only on the amino acid sequence.

```
PspA1      1     EEAPVASQSKAEKDYDAAVKKYEAAKKEYEDGKA----AQKKYEDDQKKT    46
                 EEAPVASQSKAEKDYDAA:KK.EAAKK.YE:.K.    AQKKY::.QKKT
PspA4Pro   1     EEAPVASQSKAEKDYDAAMKKSEAAKKAYEEAKKKAEDAQKKYDEGQKKT    50


PspA1      47    EEKAEEERKASEEEQAANLKYQQELVKY--IRENDPTKKAEAKKAMDEAE    94
                 EEKA.:..:AS:E...A..:.Q...VKY  ::.N....:.E.KK.:.E.:
PspA4Pro   51    EEKARKAEEASKEIAKATSEVQNAYVKYQRVQRNSRLNEKERKKQLAEID    100


PspA1      95    KEYKK-------KQTEFAEVRAKVIPSAEELKKTRQKAEEAKAKEAELTK    137
                 :E..K       K..:F.:VR.:VIP...EL.K.::KAEEAKA:E....:
PspA4Pro   101   EEINKAKQILNEKNEDFKKVREEVIPEPTELAKDQRKAEEAKAEEKVAKR    150


PspA1      138   KVEEAEKKVTEAKQKVDAEHAE----EVAPQAKIAELEHEVQKLEKALKE    183
                 K.:.A..KV..AK.KV:AE.AE    ....Q.K:A:LE.E:...EK.
PspA4Pro   151   KYDYATLKVALAKSKVEAEEAELDNKAENLQNKVADLEKEIANAEKT---    197


PspA1      184   IDESDSEDYVKEGLRAPLQFELDVKQAKLSKLEELSDKIDELDAEIAKLE    233
                                                       :.:L:.E: AKLE
PspA4Pro   198   --------------------------------------VADLEKEVAKLE    209


PspA1      234   KDVEDFKNSDGEQAGQYLAAAEEDLVA-----------------------    260
                 KDVEDFKNS:GEQA.QYLAAAE:DLVA
PspA4Pro   210   KDVEDFKNSNGEQAEQYLAAAEKDLVAKKAELAEAKIKAATKKAELEKAE    259


PspA1      261   --------------------------------------------------    260

PspA4Pro   260   AELENLLSTLDPEGKTQDELDKEAAEAELNKKVEALQNQVAELEEELSKL    309


PspA1      261   --------------------------KKAELEKTEADLKKAVNE--PEKP    282
                                           KKAELEKT:.:L..A:NE  P:..
PspA4Pro   310   EDNLKDAETNNVEDYIKEGLEEAIATKKAELEKTQKELDAALNELGPDGD    359


PspA1      283   AEETPAPAPKPEQPAEQPKPAPAPQPEKPAAEEPENPASAPQP    324
                 .EETPAPAP:PE:PA     PAPAP:P
PspA4Pro   360   EEETPAPAPQPEKPA----PAPAPKP----------------    381
```

**Fig. S2** Alignment of amino acid sequence of the untagged recombinants PspA1 and PspA4Pro The amino acid sequence of the N-terminal domain including the CDR (Red) and proline rich domain (Blue) from PspA1 and PspA4Pro were aligned The similarities are shown in the line between the sequence.

**A**

```
PspA1         MEEAPVASQSKAEKDYDAAVKKYEAAKKEYEDGKAAQKKYEDDQKKTEEKAEEERKASEEEQAANLKYQQELV
Charge        A--AAAAPPP+A-+-P-AAA++P-AA++-P--A+AAP++P---P++P--+A---++AP---PAAPA+PPP-AA
Exposure      -----------B---B--BB--B---------B---B------B--B--BB--B---------B---B--BBB

PspA1         KYIRENDPTKKAEAKKAMDEAEKEYKKKQTEFAEVRAKVIPSAEELKKTRQKAEEAKAKEAELTKKVEEAEKK
Charge        +PA+-P-AP++A-A++AA--A-+-P+++PP-AA-A+A+AAAPA--A++P+P+A--A+A+-A-AP++A--A-++
Exposure      -B-----------B---B-BB--BB--B---B--B---------BB--B---B--B---B--BB-BB--B---

PspA1         VTEAKQKVDAEHAEEVAPQAKIAELEHEVQKLEKALKEIDESDSEDYVKEGLRAPLQFELDVKQAKLSKLEEL
Charge        AP-A+P+A-A-+A--AAAPA+AA-A-+-AP+A-+AA+-A--P-P--PA+-AA+AAAPA-A-A+PA+AP+A--A
Exposure      B--B--BB------B-BB------B------B--BB--B--B--B--B---B---B--B--B---B------B

PspA1         SDKIDELDAEIAKLEKDVEDFKNSDGEQAGQYLAAAEEDLVAKKAELEKTEADLKKAVNEPEKPAEETPAPAP
Charge        P-+A--A-A-AA+A-+-A--A+PP-A-PAAPPAAAA---AAA++A-A-+P-A-A++AAP-A-+AA--PAAAAA
Exposure      ---B--B---B-----BB--B--B---B---B-BBBBB-BBB--B-B-BBBB-B--BB--B--BB--B---B-

PspA1         KPEQPAEQPKPAPAPQPEKPAEEPENPASAPQP
Charge        +A-PAA-PA+AAAAAPA-+AA--A-PAAPAAPA
Exposure      -B--BB-----------------B---------
```

**B**

```
PspA4Pro      EEAPVASQSKAEKDYDAAMKKSEAAKKAYEEAKKKAEDAQKKYDEGQKKTEEKARKAEEASKEIAKATSEVQN
Charge        --AAAAPPP+A-+-P-AAA++P-AA++AP--A+++A--AP++P--AP++P--+A++A--AP+-AA+APP-APP
Exposure      -----B----B---B--B---B--B---B--B---B--B---B------B---B------------B---B--

PspA4Pro      AYVKYQRVQRNSRLNEKERKKQLAEIDEEINKAKQILNEKNEDFKKVREEVIPEPTELAKDQRKAEEAKAEEK
Charge        APA+PP+AP+PP+AP-+-+++PAA-A---AP+A+PAAP-+P--A++A+--AAA-AP-AA+-P++A--A+A--+
Exposure      B-B-B--B-----B-----------B---B--B--BB------B--BB--BB-----BB-----B--B----B

PspA4Pro      VAKRKYDYATLKVALAKSKVEAEEAELDNKAENLQNKVADLEKEIANAEKTVADLEKEVAKLEKDVEDFKNSN
Charge        AA+++P-PAPA+AAAA+P+A-A--A-A-P+A-PAPP+AA-A-+-AAPA-+PAA-A-+-AA+A-+-A--A+PPP
Exposure      -B---B-BBBB--B----BB------B---B--B---B--B---B--B---BB-BB--B---B--B-------

PspA4Pro      GEQAEQYLAAAEKDLVAKKAELAEAKIKAATKKAELEKAEAELENLLSTLDPEGKTQDELDKEAAEAELNKKV
Charge        A-PA-PPAAAA-+-AAA++A-AA-A+A+AAP++A-A-+A-A-A-PAAPPA-A-A+PP--A-+-AA-A-AP++A
Exposure      B--B--BB--B--BB------B--B---B--B---B--BB--B--B---B-----B---B------B-B---B

PspA4Pro      EALQNQVAELEEELSKLEDNLKDAETNNVEDYIKEGLEEAIATKKAELEKTQKELDAALNELGPDGDEEETPA
Charge        -AAPPPAA-A---AP+A--PA+-A-PPPA--PA+-AA--AAAP++A-A-+PP+-A-AAAP-AAA-A----PAA
Exposure      --B---B--B---B--B---B--B----B---B---B--BB----------B--B-BBB--B-----------

PspA4Pro      PAPQPEKPAPAPAPKP
Charge        AAAPA-+AAAAAAA+A
Exposure      B---B-----------
```

**C**

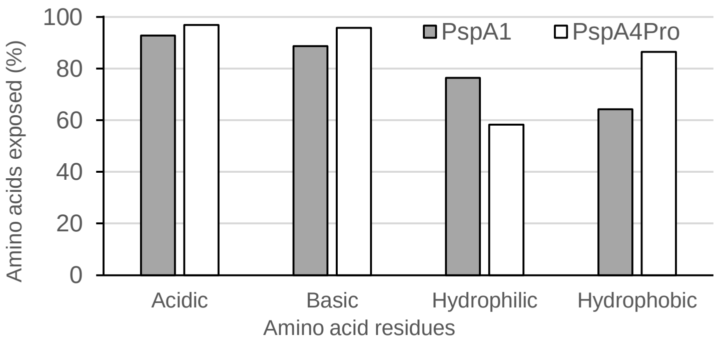


**Fig. S3** Analysis of charge and exposure of amino acids of untagged recombinants PspA1 and PspA4Pro. The PspA1 (A) and PspA4Pro (B) amino acid sequences of the N-terminal and proline rich domains (Black) were aligned with the charge (Blue) and the position on the surface (Red) of each amino acid. The percentage of exposed amino acids on the surface (C) of both proteins were also calculated. In charge sequence - means negative charged amino acid; + means positive charged amino acid; P means polar amino acid; and A means apolar amino acid. In exposure analysis - means prediction of exposure of amino acid on the surface; B means prediction of buried amino acid.

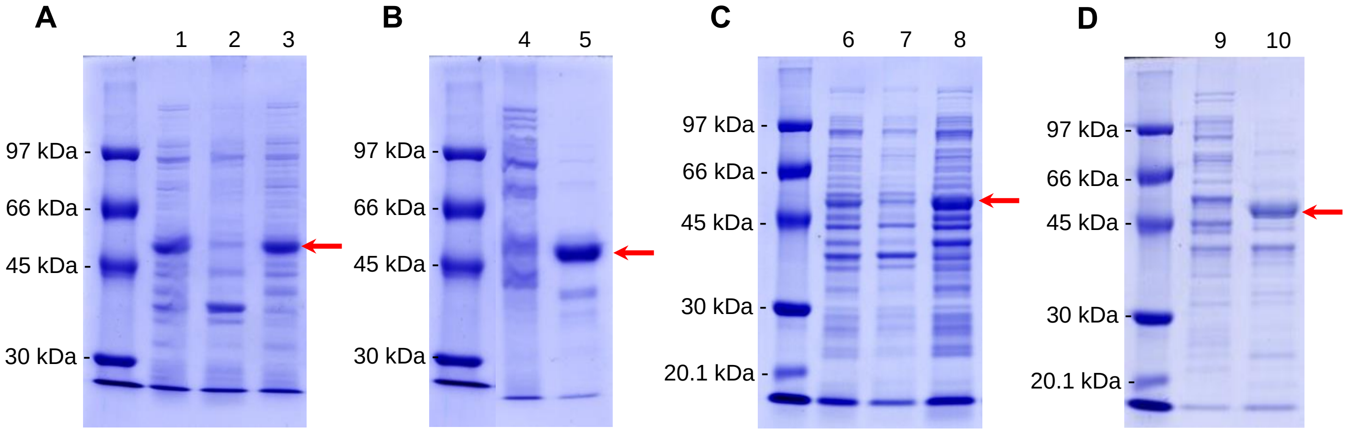


**Fig. S4 S4** Recovery of PspA1 in the soluble fraction. The 80 g of biomass was used for the final purification process (A and B) and the replicate of that (C and D). The Homogenate fraction from the lysis process (1 and 6), followed by treatment with CTAB produced a insoluble fraction (2 and 7) and soluble fraction (Clarified fraction) (3 and 8) containing the protein PspA1. The Clarified fraction was then applied to the AEC for recover of PspA1 with 250 mM NaCl. Then, the 250 mM NaCl fraction was cryoprecipitated and generated a insoluble fraction (4 and 9) and a soluble fraction (Cryoprecipitate, pH 4.0) (5 and 10). The protein content in each sample was quantified by the Lowry colorimetric method. The samples were loaded into the gel by using a total amount of protein in each lane of 10 μg (1-3 and 6-8), 5 μg (4-5 and 9-10). The red arrows indicate the PspA1 protein.
